# Heterologous Ectoine Production in *Escherichia coli*: Optimization Using Response Surface Methodology

**DOI:** 10.1155/2019/5475361

**Published:** 2019-07-01

**Authors:** I Putu Parwata, Deana Wahyuningrum, Sony Suhandono, Rukman Hertadi

**Affiliations:** ^1^Biochemistry Research Division, Faculty of Mathematics and Natural Sciences, Institut Teknologi Bandung, Bandung, Indonesia; ^2^Analytical Chemistry Department, Faculty of Mathematics and Natural Sciences, Universitas Pendidikan Ganesha, Denpasar, Indonesia; ^3^Organic Chemistry Research Division, Faculty of Mathematics and Natural Sciences, Institut Teknologi Bandung, Bandung, Indonesia; ^4^Genetics and Molecular Biology Division, School of Biological Science and Technology, Institut Teknologi Bandung, Bandung, Indonesia

## Abstract

**Introduction:**

A halophilic bacterium of the *Halomonas elongata* BK-AG25 has successfully produced ectoine with high productivity. To overcome the drawbacks of high levels of salt in the production process, a nonhalophilic bacteria of *Escherichia coli* (*E. coli*) was used to express the ectoine gene cluster of the halophilic bacteria, and the production of ectoine by the recombinant cell was optimized.

**Methods:**

The ectoine gene cluster from the halophilic bacterium was isolated and inserted into an expression plasmid of pET30(a) and subsequently transformed into *E. coli* BL21 (DE3). Production of ectoine from the recombinant *E. coli* was investigated and then maximized by optimizing the level of nutrients in the medium, as well as the bioprocess conditions using response surface methodology. The experimental designs were performed using a central composite design.

**Results:**

The recombinant *E. coli* successfully expressed the ectoine gene cluster of *Halomonas elongata* BK-AG25 under the control of the *T7* promoter. The recombinant cell was able to produce ectoine, of which most were excreted into the medium. The optimization of ectoine production with the response surface methodology showed that the level of salt in the medium, the incubation temperature, the optical density of the bacteria before induction, and the final concentration of the inducer gave a significant effect on ectoine production by the recombinant *E. coli*. Interestingly, the level of salt in the medium and the incubation temperature showed an inverse effect on the production of intracellular and extracellular ectoine by the recombinant cell. At the optimum conditions, the production yield was about 418 mg ectoine/g cdw (cell dry weight) after 12 hours of incubation.

**Conclusion:**

This study is the first report on the expression of an ectoine gene cluster of *Halomonas elongata* BK-AG25 in *E. coli* BL21, under the control of the *T7* promoter. Optimization of the level of nutrients in the medium, as well as the bioprocess condition using response surface methodology, has successfully increased the production of ectoine by the recombinant bacteria.

## 1. Introduction

Osmotic stress has forced halophilic microorganisms to develop two different strategies to survive in a salty habitat. First, using salt-in-cytoplasm strategy, the microorganism accumulates potassium ions, K^+^, and their counterion (mainly glutamate), in the cytoplasm. Second, in the organic-osmolyte strategy, organic-compatible molecules such as glycine, betaine, ectoine, hydroxyectoine, trehalose, and proline are imported by the microbes to balance the osmotic pressure outside the cell [1]. Ectoine (1,4,5,6-tetrahydro-2-methyl-4-pyrimidine carboxylic acid) is one of the widely used, compatible osmolytes produced by halophilic microorganisms. Besides an osmotic protector, ectoine has also been shown to possess the ability to counter various environmental stresses, such as heating, freezing, drought, UV light, and contact with toxic materials [2–4].

The use of ectoine as a bioactive compound in pharmaceuticals products and cosmetics leads to the increasing commercial demand of this valuable molecule. In cosmetics, ectoine is widely used as a skin protector against dehydration and UV light, as well as found in antiaging and moisturizing agents [5]. As a consequence, a number of efforts have been conducted to improve ectoine production in microorganisms. One of the most popular methods is the production of ectoine from the halophilic bacteria *Halomonas elongata*, using “bacterial milking” processes [6]. This method has successfully manufactured ectoine on a large scale. However, the high level of salt used in this conventional method has resulted in corrosion of equipment, reduction of cell growth rate, and difficulty of downstream processing [7].

To overcome the drawbacks of the conventional methods, the use of transgenic nonhalophilic bacteria has improved the production of ectoine. Ectoine biosynthetic gene clusters from different halophiles had been expressed in *Escherichia coli* (*E. coli*), and ectoine was successfully produced by the recombinant *E. coli* [7–11]. Interestingly, under the control of nonsalt-inducible promoters, most of the ectoines produced by the recombinant *E. coli* were excreted into the medium. In contrast, when using salt-sensitive regulatory promoters indigenous to halophiles, most ectoines remained intracellularly [7, 9].

In a previous study, we have isolated a halophilic bacterium of the *Halomonas elongata* BK-AG25 strain from the mud crater of Bledug Kuwu, located in Kuwu Village, Kradenan District, Grobogan Regency, Central Java, Indonesia. The bacteria obtained from this unique crater were able to produce ectoine with high productivity. However, the production of ectoine by the bacteria was carried out using a medium containing a high level of salt, which caused problems on the equipment and downstream processing. Hence, in the present study, we used a nonhalophilic bacteria of *E. coli* to express the ectoine gene cluster of *Halomonas elongata* BK-AG25, under a strong *T7* promoter. The level of salt and glucose in the medium, the incubation temperature, the optical density (OD) of the bacterial culture before induction, and the final concentration of the inducer were optimized for ectoine production by the recombinant *E. coli*, using response surface methodology (RSM). RSM is a statistical approach for evaluating the relationship between factors and responses, as well as determining the optimum conditions for the investigated responses [12]. In RSM, the relationship between factors and responses is efficiently studied without involving a large number of experimental trials [13]. This statistical approach is used widely to optimize critical factors in biotechnological processes [14–18]. We found here that most ectoines produced by the recombinant *E. coli* were excreted into the medium. The inverse effect of the salt level and the incubation temperature on ectoine production by the recombinant *E. coli* is discussed.

## 2. Methods

### 2.1. Experimental Design

The optimizations of ectoine production by the recombinant *E. coli* were performed in two steps using response surface methodology. The first was conducted to optimize the level of glucose (0.3–1.7% (w/v)) and NaCl (0.1–2.7% (w/v)) in MM63 medium and the incubation temperature (15–45°C), followed by the second step to optimize the optical density (OD_600_) of the bacterial culture before induction (0.3–1.2) and the final concentration of isopropyl *β*-D-1-thiogalactopyranoside (IPTG) as inducer (0.1–1.5 mM). The central composite design (CCD) was used to obtain a five-level range of each parameter optimized (Table 1).

*Halomonas elongata* BK-AG25 samples were isolated from a salty mud crater located in Bleduk Kuwu village, Central Java, Indonesia. The bacteria were maintained in Luria Bertani (LB) media containing (w/v): 1% tryptone, 0.5% yeast extract, 10% NaCl, and 2% Bacto agar. *E. coli* TOP 10 was used in the construction of recombinant plasmids, while *E. coli* BL21 (DE3) pLysS (F–, *omp*T, *hsd*S_B_ (r_B_–, m_B_–), *dcm*, *gal*, *λ* (DE3), pLysS, Cm^r^) was used as the host for protein expression and ectoine production. *E. coli* was grown in LB medium with the composition of (w/v): 1% tryptone, 0.5% yeast extract, and 1% NaCl at 37°C for 15 hours. Expression of protein and production of ectoine by the recombinant *E. coli* were performed using MM63 medium [19] composed of (w/v): 1.36% KH_2_PO_4_, 0.421% KOH, 0.198% (NH_4_)_2_SO_4_, 0.025% MgSO_4_·7H_2_O, 0.00011% FeSO_4_·7H_2_O, 0.5% glucose·H_2_O, and 1% NaCl. The antibiotic kanamycin (final concentration of 50 *μ*g/mL) was used to maintain the heterologous plasmid in the genetically modified strain.

### 2.2. DNA Manipulation and Construction of Plasmid

Standard methods were used for the isolation of genomic DNA, plasmid construction, and transformation of cell host [20]. The ectoine gene cluster (*ectABC*) was amplified from the genomic DNA of *Halomonas elongata* BK-AG25 using polymerase chain reaction (PCR). Two primers containing NdeI and EcoRI restriction sites were used to attain the gene cluster as follows: Ect1 5′-*CATATG*AACGCAACCACAGAGCC-3′ and Ect2 5′-*GAATTC*CGGGTTACAGCGGCTTC-3′ (NdeI and EcoRI sites are in italics). The *ectABC* gene was amplified without any upstream operator/promoter and downstream terminator region and was verified by PCR product sequencing. The PCR products were ligated into the cloning vector pGEM-T Easy (Promega) and transformed into *E. coli* TOP 10. The cloning vector containing the *ectABC* genes was isolated from the recombinant *E. coli* TOP 10 and digested with NdeI and EcoRI. The NdeI-EcoRI fragments were subsequently recloned into the expression plasmid pET-30a(+) (Invitrogen, USA) under the control of the *T7* promoter. The recombinant plasmid harboring the *ectABC* genes was verified by plasmid sequencing. The resulting plasmid pET-ectABC was transformed into *E. coli* BL21 (DE3). The recombinant *E. coli* harboring the *ectABC* genes was then verified using colony PCR.

### 2.3. Protein Expression

*E. coli* BL21 harboring the recombinant plasmid pET-ectABC were grown at 37°C in LB broth containing kanamycin for 15 hours. Subsequently, 2% (v/v) of the bacterial culture was inoculated in fresh LB broth and incubated at 37°C for 2 hours until the culture reached around 0.5 optical density at 600 nm (OD_600_). IPTG (isopropyl *β*-D-1-thiogalactopyranoside) was then added into the bacterial culture at a final concentration of 0.5 mM and incubated at 30°C for 4 hours. The cells were harvested by cold centrifugation at 8,000 × g for 10 minutes and resuspended in 50 mM of potassium phosphate buffer (pH 7.0). The cell suspension was then sonicated (Sonic Vibra Cell, USA) at a frequency of 40 Hz for 15 minutes and centrifuged at 12,000 × g for 10 minutes. The supernatant was then subjected to sodium dodecyl sulfate-polyacrylamide gel electrophoresis (SDS-PAGE) analysis.

### 2.4. SDS-PAGE Analysis

The supernatant was mixed with loading buffer (composition: 125 mM Tris-HCl, pH 6.8, 20% glycerol, 4% SDS, 10% *β*-mercaptoethanol, and 0.5 mg/mL bromophenol blue) in a ratio of 4 : 1 and then boiled in water for 5 minutes to denature proteins. After centrifugation at 10,000 × g for 1 minute, 20 *μ*L of suspension was loaded into polyacrylamide gel. Subsequently, electrophoresis was run at 120 V for 80 minutes. The gel was then removed and stained for 12 hours with 100 mL of staining solution containing 45 mL methanol, 45 mL ddH_2_O, 10 mL glacial acetic acid, and 0.25 g Coomassie brilliant blue. The unbound solute was then removed for more than 4 hours using 100 mL of the destaining solution comprising 45 mL methanol, 45 mL ddH_2_O, and 10 mL glacial acetic acid [20].

### 2.5. Production and Extraction of Ectoine

The recombinant *E. coli* BL21 were grown in MM63 medium at 37°C for 15 hours, subsequently transferred into fresh MM63 medium, and incubated in a shaker at 37°C for 4 hours until OD_600_ of the culture reached around 0.5. IPTG was then added into the bacterial culture at a final concentration of 0.5 mM and incubated at 30°C for 14 hours for ectoine production. Afterward, the samples were taken for biomass and ectoine analysis. The bacterial culture was cold centrifuged at 8,000 × g for 10 minutes, and the extracellular ectoine in the supernatant and the intracellular ectoine in the bacterial cell were then determined.

The intracellular ectoine produced by the recombinant cell was extracted from the cell following the procedures presented by Bligh and Dyer [21]. First, the cells were separated from 1 mL of a bacterial culture by centrifugation at 8,000 × g for 10 minutes at 4°C and lyophilized. The dried cells were then extracted by vigorous shaking for 60 minutes in 400 *μ*L mixture of methanol/chloroform/water (10/5/4 (vol/vol/vol)). Subsequently, an equal volume (130 *μ*L each) of chloroform and water were added and then stirred for 30 minutes. After centrifugation at 10,000 × g for 30 minutes, the water phase containing the ectoine was lyophilized and resuspended in water. The extracellular ectoine excreted by the bacteria into the medium was directly analyzed.

### 2.6. Analytical Methods

Biomass concentration was determined following the procedure proposed by Van-Thuoc et al. [18]. After cold centrifugation at 8,000 × g for 10 minutes, the cells were washed with media (without glucose) and dried at a temperature of 70°C to obtain a constant weight. The biomass concentration is expressed as a gram of cell dry weight (cdw) per liter of bacterial culture.

The ectoine solution was subjected to 0.2 *μ*m filter membrane for high-performance liquid chromatography (HPLC) analysis. Ectoine was determined using Agilent Technologies 1260 Infinity HPLC (Germany) on a Nucleosil 100–5 C18, 25.0 cm by 3.2 mm (5 *μ*m) column (Sigma-Aldrich, USA). Twenty microliters of the sample was injected to the column, and ectoine was monitored by its absorbance at 210 nm using an ultraviolet/visible (UV/VIS) detector. Separation of solutes was conducted isocratically using water/acetonitrile (95/5 [vol/vol]), at a flow rate of 1 mL/min at 20°C. The ectoine retention time was determined using standards purchased from Sigma-Aldrich.

### 2.7. Statistical Analysis

The concentrations of ectoine produced by the recombinant *E. coli* and the bacterial productivities were fitted into a full quadratic model using Minitab17 software. The fitting of the models was justified using analysis of variance (ANOVA). The significance of each regression coefficient was tested using Student's *t*-test, with a confidence level of 5%.

## 3. Results and Discussion

### 3.1. Cloning and Expression of Ectoine Gene Cluster in *E. coli*

**Figure 1:**
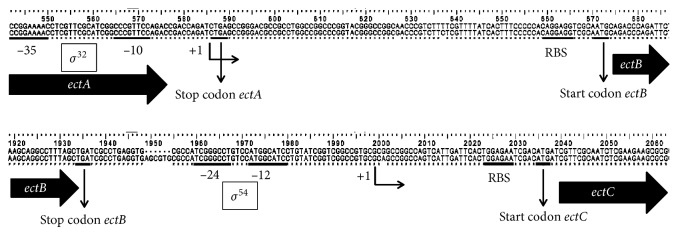
Sequences of the promoter and ribosomal binding site (RBS) on *ectB* and *ectC* genes of *Halomonas elongata*. Upper sequence: *Halomonas elongata* DSM2581; lower sequence: *Halomonas elongata* BK-AG25 (this study).

The sequence of the ectoine gene cluster (*ectABC*) of *Halomonas elongata* BK-AG25 was consistent with the sequences reported. The gene is 2,438 base pairs (bp) in length, composed of *ectA* (579 bp), *ectB* (1,266 bp), and *ectC* (414 bp). Using BioEdit version 7.0.9.0, an alignment analysis of the gene with the closest related gene of the *Halomonas elongata* DSM2581 strain showed conserved region of internal promoter and ribosomal binding site (Figure 1). The sequences at −10 (CCCGTTCC) and −35 (CCGGAAA) upstream of *ectB* are recognized by the heat-shock factor *σ* ^32^, as reported in [22]. Furthermore, Schwibbert et al.[23] reported that the upstream region of *ectC*, located at −12 (ATGGCAT) and −24 (TCGGGCCT), is a typical promoter of factor *σ* ^54^, which is involved in the transcription of nitrogen-regulated genes [24, 25].

**Figure 2:**
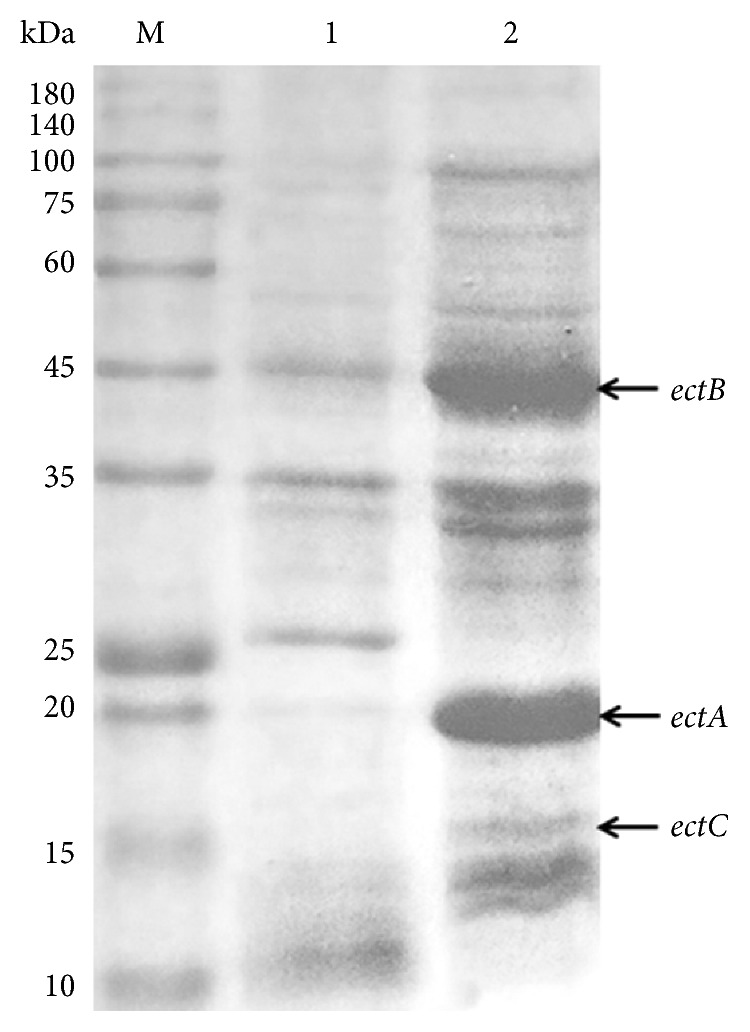
SDS-PAGE analysis of *ectA*, *ectB*, and *ectC* expressed in *E. coli*. M: protein molecular mass marker; lane 1: supernatant of cell extracts from uninduced recombinant *E. coli*; lane 2: supernatant of cell extracts from induced recombinant *E. coli*.

The expression of the ectoine gene cluster (*ectABC*) of *Halomonas elongata* BK-AG25 in *E. coli* resulted in three clear bands, with the estimated molecular mass of about 21 kDa, 45 kDa, and 16 kDa as revealed by SDS-PAGE analysis (Figure 2). Those bands correspond, respectively, to *ectA*, *ectB*, and *ectC* from *Halomonas elongata*, suggesting that the gene cluster was successfully expressed in *E. coli* BL21. However, the expression level of the *ectC* gene was lower than that of *ectA* and *ectB*, as evident by the thin band at about 16 kDa on the SDS-PAGE electropherogram (Figure 2).

The factors influencing the low expression of *ectC* were not yet understood. The aligned spacing between the Shine-Dalgarno (SD) sequence and the start codon AUG played an important role in initiating the translation process. Chen et al. [26] reported that the aligned spacing between 4 and 6 nucleotides resulted in high expression of chloramphenicol acetyltransferase in *E. coli*. In our study, the aligned spacing between SD (underlined) and AUG (bold) of *ectA* (AAGGAGAUAUACAU**AUG**), *ectB* (AGGAGGUCGCA**AUG**), and *ectC* (GGAGAAUCGAC**AUG**) were 6, 4, and 5 nucleotides, respectively, suggesting that the low expression of EctC was not due to this factor.

**Figure 3:**
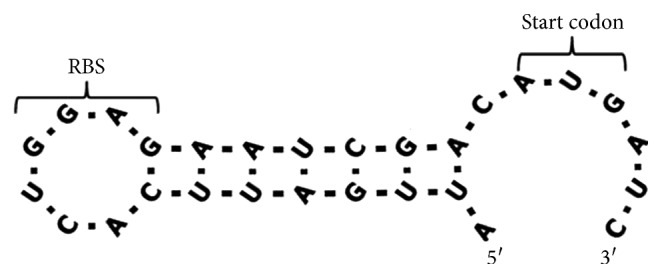
Predicted hairpin structure of the translation initiation region of *ectC* of *Halomonas elongata* BK-AG25. Ribosomal binding site (RBS) and start codon of *ectC* gene are pointed.

The secondary structure of the mRNA upstream of the start codon was reported to have a negative effect on the initiation of the translation process, due to evidence that ribosomes strongly bind single-stranded RNA [27–29]. De Smit and Duin [30] showed that the relative expression of their studied gene declined significantly when the hairpin structure in the initiation region of translation was strengthened by mutation. We have inspected the secondary structure of the initiation region of *ectABC* of *Halomonas elongata* BK-AG25 using Vienna RNA Web Services (Institute for Theoretical Chemistry). The results showed that the initiation region of *ectC* tends to form a hairpin structure with a minimum free energy of −5.70 kcal/mol (Figure 3). However, this secondary structure was not found in the initiation region of *ectA* and *ectB*, suggesting that the low expression level of *EctC* was probably caused by this hairpin structure.

### 3.2. Production of Ectoine by the Recombinant *E. coli*

**Figure 4:**
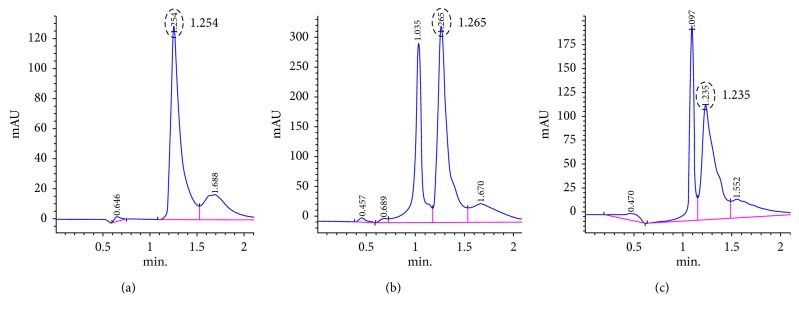
HPLC analysis of authentic ectoine and ectoines produced by the recombinant *E. coli*. (a) Authentic ectoine (Fluka, Sigma). (b) Intracellular ectoine. (c) Extracellular ectoine. Ectoine is confirmed by the peak at the retention time of about 1.2 minutes (circle).

Ectoine was successfully produced and excreted by the recombinant *E. coli*. The analysis of the intracellular and extracellular ectoine using HPLC resulted in a new peak at the same retention time as the authentic ectoine, i.e., around 1.2 minutes (Figure 4). However, other peaks at the retention time of around 1 minute were also detected in both the intracellular and extracellular ectoine. These peaks possibly correspond to *N*-*γ*-acetyldiaminobutyric acid (ADABA), which is the intermediate compound for the last step in ectoine biosynthetic pathway. This was confirmed by proton nuclear magnetic resonance spectroscopy (^1^H-NMR) analysis, which showed new peaks of an aliphatic compound corresponding to ADABA (data not shown). The abundance of ADABA was caused by the low expression of ectoine synthase (*ectC*), which is responsible for the cyclic condensation of ADABA to ectoine.

Most of the ectoines synthesized by the recombinant *E. coli* were excreted into the medium (Table 2). After induction using IPTG, the recombinant cells produced a total of 0.3 g/L ectoine, of which 0.23 g/L were excreted into the medium. Only about 0.07 g/L ectoine remained in the bacterial cells. These results showed that up to 77% of ectoine was excreted into the medium. The percentage of ectoine excreted by the recombinant *E. coli* in this study was fairly lower than the one reported by [9] which showed that under arabinose-induced promoter, the expression of ectoine gene cluster of *Halomonas elongata* in *E. coli* K-12 strain BW25113 could produce and excrete more than 90% of ectoine into the medium.

### 3.3. Optimization of the Level of Glucose and Salt in the Medium and the Incubation Temperature for Ectoine Production by the Recombinant *E. coli*

The first optimization of ectoine production by the recombinant *E. coli* was conducted on three factors: the level of glucose, salt (NaCl) in the medium, and the incubation temperature. The production of ectoine was conducted by adding 0.5 mM IPTG into the recombinant culture at an initial optical density (OD_600_) of around 0.5. The results showing the intracellular and extracellular ectoine concentration produced by the recombinant cells are presented in Supplementary Tables 1 and 2. The analysis of the experimental data resulted in response models for intracellular and extracellular ectoine concentration, with a high coefficient of determination (*R* ^2^) of 0.91 and 0.87, respectively, indicating a good agreement between the experimental data and the predicted value. Furthermore, the lack of fit values for both models is insignificant (*p* value of 0.208 and 0.512 for intracellular and extracellular ectoine concentration, respectively). Thus, the experimental data are very fit for the models. The regression equations in noncoded units for intracellular and extracellular ectoine are given below and used to calculate the predicted values of both (data are shown in Supplementary Tables 1 and 2):

(1) $$\begin{array}{c} \text{intracellular ectoine}\left(\frac{g}{L}\right)=-0.042+0.0148X_{2}+0.00521X_{3}-0.01815X_{2}\times X_{2}-0.000123X_{3}\times X_{3}+0.00154X_{2}\times X_{3}, \end{array}$$

(2) $$\begin{array}{c} \text{extracellular ectoine}\left(\frac{g}{L}\right)=-0.325+0.214X_{1}+0.0229X_{2}+0.03262X_{3}-0.1155X_{1}\times X_{1}-0.0413X_{2}\times X_{2}-0.000477X_{3}\times X_{3}. \end{array}$$

The regression coefficients of both response models and their significance test results are shown in Table 3. Our study revealed that NaCl content in the medium and the incubation temperature had a significant effect on intracellular and extracellular ectoine produced by the recombinant *E. coli* (*p* value < 0.05). Meanwhile, the glucose levels in the medium (in the range of 0.3–1.7% w/v) did not show a significant effect in both intracellular and extracellular ectoine production.

The salt content in the medium provided different linear effects for intracellular and extracellular ectoine concentration. The salt level showed a strong negative linear effect on extracellular ectoine (regression coefficient of −0.07172) but gave a positive linear effect on intracellular ectoine (regression coefficient of 0.00786). These results indicate that elevated levels of salt in the medium will decrease the extracellular ectoine but increase the intracellular ectoine concentration, and vice versa. High levels of salt outside the cells trigger the recombinant cells to synthesize and store more ectoine in the cytoplasm, in order to balance the osmotic pressure outside the cell. In contrast, when the levels of salt outside the cell are reduced, the ectoine produced by the bacteria will instead be excreted to avoid excessive water flow into the cell. These findings are supported by [31, 32], who stated that when the environmental stress is increased, the bacteria will increase the number of osmolytes stored in the cells but release them when the environmental stress is decreased. This phenomenon is widely used to optimize ectoine production using the osmotic shock technique [6, 18]. At first, bacteria are grown in the medium containing high levels of salt (osmotic upshock) to stimulate the biosynthesis of ectoine. The cells are then transferred to the medium containing lower levels of salt (osmotic downshock) to force the bacteria to excrete ectoine into the medium.

**Figure 5:**
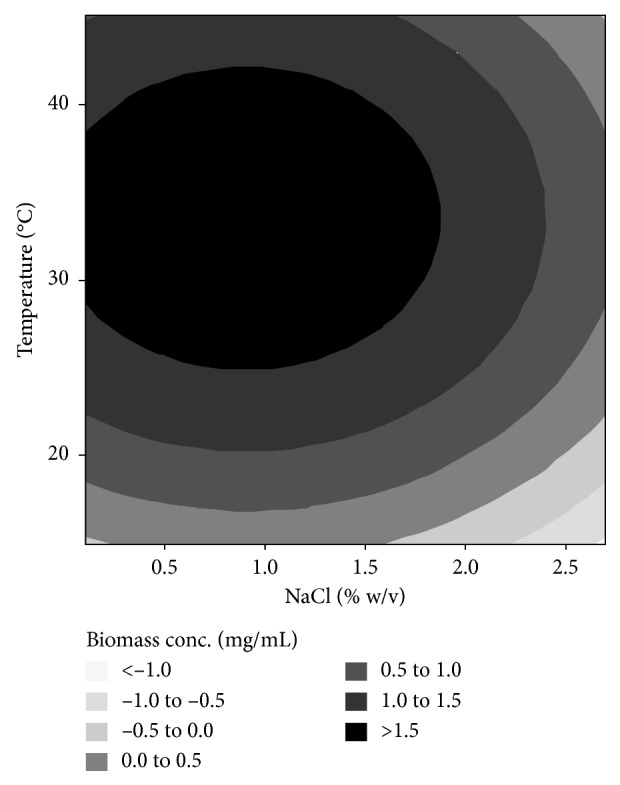
Effect of salt level in the medium and incubation temperature on biomass concentration of the recombinant *E. coli*.

The effect of the incubation temperature on ectoine production by the recombinant *E. coli* was inversely proportional to the effect of the salt level. The incubation temperature showed a positive linear effect on extracellular ectoine concentration (regression coefficient of 0.03565) but gave a very weak and insignificant negative linear effect on intracellular ectoine (regression coefficient of −0.00015). Based on these results, an increase in incubation temperature gave an increase in extracellular ectoine concentration. This was due to high biomass concentration of the bacteria at relatively high temperatures, as shown in Figure 5. The increase in biomass is proportional to the increase in ectoine yield as the biosynthesis of ectoine is conducted intracellularly. Ectoine and other osmolytes are accumulated inside the cells to counter the osmotic stress outside the cells [1, 33].

**Figure 6:**
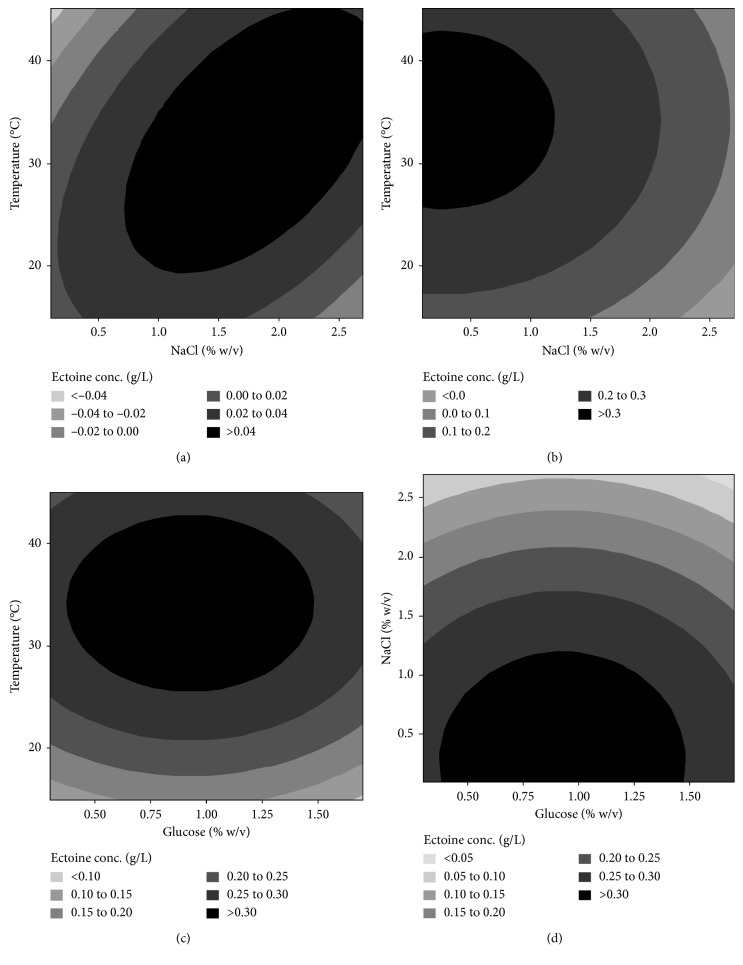
Effect of nutrients in MM63 medium and incubation temperature on the concentration of intracellular ectoine (a) and extracellular ectoine (b, c, and d) produced by the recombinant *E. coli*.

The response model for intracellular ectoine showed a strong interaction between the salt content in the medium and the incubation temperature (interaction coefficient of 0.01042 with a *p* value of 0.000). The combination of high NaCl content and low incubation temperature, or high incubation temperature and low NaCl content, led to decreases in intracellular ectoine produced by the recombinant cell (Figure 6(a)). Based on the response model obtained, the optimal intracellular ectoine concentration was reached when the level of NaCl in the medium was in the range of 0.8–2.7% (w/v) and at a temperature range of 20–45°C (Figure 6(a)). Meanwhile, the optimal extracellular ectoine was obtained when the recombinant cells were incubated in the medium containing glucose levels of 0.5–1.4% (w/v) and NaCl levels of 0.1–1.0% (w/v), at temperatures of 25–42°C (Figures 6(b)–6(d)).

The maximal extracellular ectoine concentration of 0.34 g/L was predicted by the regression model when the recombinant *E. coli* were incubated in MM63 medium containing 0.92% (w/v) glucose and 0.28% (w/v) NaCl at 34°C. The experimental results under these conditions attained an average concentration of extracellular ectoine of 0.37 ± 0.027 (standard error) g/L (data are shown in Supplementary Table 2). Meanwhile, the intracellular ectoine concentration of 0.055 g/L was predicted by the model at the optimum conditions of 1.78% (w/v) NaCl and 32°C. Under these conditions, the intracellular ectoine concentration had an average of 0.050 ± 0.005 g/L (data are shown in Supplementary Table 1). The experimental data are quite close to the predicted value of the response model.

**Figure 7:**
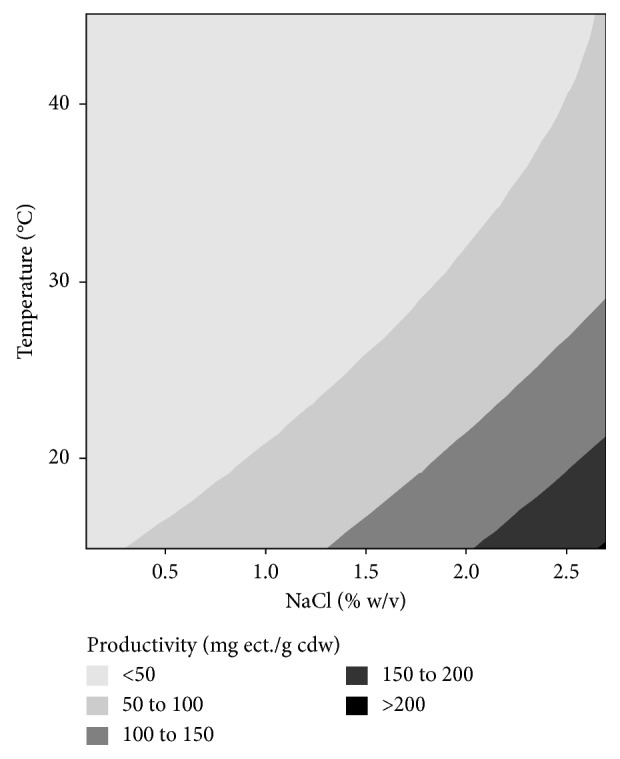
Effect of NaCl level in MM63 medium and incubation temperature on the productivity of the recombinant *E. coli* to produce intracellular ectoine.

The experimental data on productivity of recombinant *E. coli* to produce intracellular ectoine (shown in Supplementary Table 3) generated a regression model with determination factor value (*R* ^2^) of 0.98, indicating that 98% of variation in the regression model was determined by experimental data. The significance test of regression coefficient using Student's *t*-test is shown in Table 3. The salt level and incubation temperature showed significant effect on the productivity of the recombinant cell (*p* value < 0.05), but not significant for the glucose level. Both factors had an inverse effect on bacterial productivity. The salt level gave a strong positive effect (coefficient value of 27.55), while the incubation temperature showed a strong negative effect (coefficient value of −24.88). These results indicate that the productivity of the recombinant cells was optimal at high NaCl levels (above 2.0% w/v) and at low incubation temperature (below 20°C) (Figure 7). These findings are consistent with [34], who stated that the three enzymes in ectoine biosynthesis, diaminobutyric acid (DABA) aminotransferase, DABA acetyltransferase, and ectoine synthase showed optimum activity at low temperatures of 10–25°C and high levels of NaCl (2.3–2.9% w/v). However, the concentration of ectoine produced at those conditions was very low (Figure 6(a)), due to the low biomass yield of the bacteria (Figure 5).

### 3.4. Optimization of the Bacterial Optical Density (OD) before Induction and the Final Concentration of the Inducer for Ectoine Production by the Recombinant E. coli

The second optimization of the ectoine production by the recombinant *E. coli* was performed on two factors: the optical density (OD) of the bacterial culture before induction and the final concentration of the inducer (IPTG). The results of the extracellular ectoine concentration and the productivity of the bacteria to produce extracellular ectoine are shown in Supplementary Tables 4 and 5. The regression model generated by RSM analysis on both data was very convincing, with determination coefficient (*R* ^2^) values of 88.52% and 92.05% for extracellular ectoine concentration and productivity of the recombinant cell, respectively. The fitting of the models was also supported by the lack of fit values for both models, which are insignificant (*p* values of 0.30 and 0.27 for extracellular ectoine concentration and productivity of the bacteria, respectively). Therefore, both models can be used to predict the levels of ectoine and the productivity of the recombinant cells using the regression equations as follows (predicted results are shown in Supplementary Tables 4 and 5).

(3) $$\begin{array}{c} \text{extracellular ectoine}\left(\frac{g}{L}\right)=0.113+1.103X_{1}+0.547X_{2}\;-0.549X_{1}\times X_{1}-0.1624X_{2}\times X_{2}-0.467X_{1}\times X_{2}, \end{array}$$

(4) $$\begin{array}{c} \text{productivity}\left(\frac{mg\;ect.}{g\text{ cdw}}\right)=220.4+131.1X_{1}+219.0X_{2}-59.5X_{1}\times X_{1}-52.2X_{2}\times X_{2}-201.1X_{1}\times X_{2}. \end{array}$$

Table 4 shows the significance test results of each regression coefficient for both models, using Student's *t*-test with a significance level of 5%. The OD of the bacterial culture before induction and the final concentration of the inducer (IPTG) showed significant negative linear effect on extracellular ectoine produced by the recombinant *E. coli* (*p*value < 0.05). The strength of these two factors was equivalent in affecting extracellular ectoine production, as indicated by the equivalent linear regression coefficient values of −0.0301 and −0.0313 for OD value and IPTG concentration, respectively. However, the productivity of the bacteria was only affected by the value of bacterial OD before induction. As the productivity of the bacteria is defined as the amount of ectoine per cell dry weight, this is thus significantly dependent on the density of the cell (cell growth), which in turn is affected by the initial OD of the bacterial culture. The concentration of the inducer (IPTG), in the optimized range of 0.1–1.5 mM, did not give a linear effect on bacterial productivity, indicating that the amount of ectoine produced wsa greatest at the minimum concentration of the inducer (0.1 mM). As a consequence, the productivity of the bacteria was predominantly influenced by the density of the cell.

**Figure 8:**
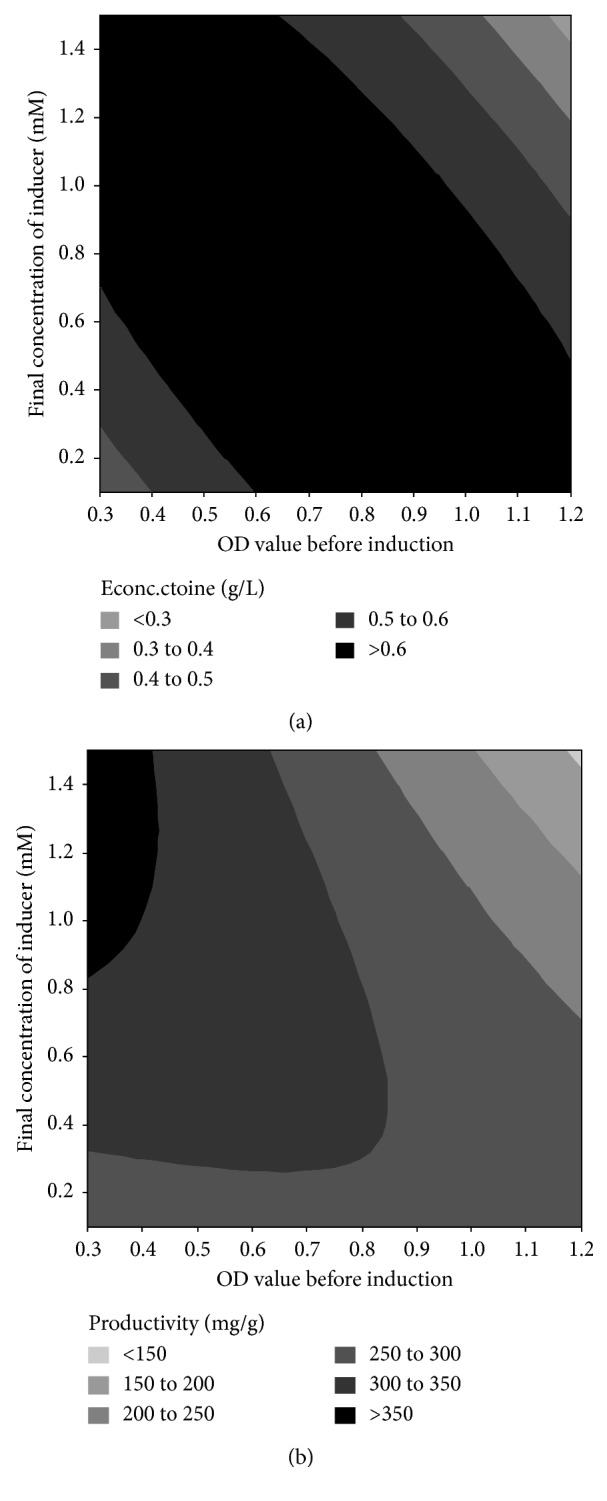
Effect of OD value before induction and the final concentration of the inducer on ectoine production by the recombinant *E. coli.* (a) The concentration of ectoine. (b) The productivity of the recombinant *E. coli*.

The regression model showed a strong interaction between the OD value before induction and the final concentration of the inducer, by influencing the concentration of ectoine produced by the recombinant *E. coli* (interaction coefficient of −0.0736 with a *p* value of 0.004). Induction of the recombinant cells at high initial OD (above 1.1) using the high final concentration of IPTG (above 1.2 mM) led to a decline of extracellular ectoine concentration. Similarly, a combination of low initial OD value (below 0.4) and low IPTG concentration (below 0.3 mM) decreased the production of extracellular ectoine. Thus, to obtain the optimal concentration of extracellular ectoine, bacterial culture with a low or high initial OD value should be induced with a high or low concentration of IPTG, respectively (Figure 8(a)). The induction time, which is correlated with the initial OD of the bacterial culture and the concentration of the inducer, has a significant effect on the expression of the protein in the recombinant cell [35, 36]. An appropriate time of induction, as well as the concentration of the inducer, will result in an optimum yield of soluble protein, which is the main goal of the expression of genes.

**Figure 9:**
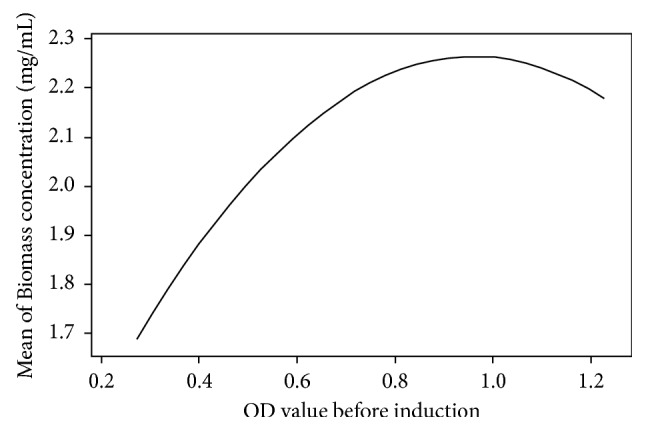
Effect of OD value before induction on biomass concentration of the recombinant *E. coli*.

The regression model for the productivity of the recombinant *E. coli* to produce extracellular ectoine showed different patterns in the optimum region (Figure 8(b)) compared to the model for extracellular ectoine concentration (Figure 8(a)). The optimum bacterial productivity was obtained at low initial OD value (<0.45) and high concentration of IPTG (>0.8 mM). This can be explained by inspecting the effect of initial OD values on the concentration of biomass produced by the bacteria after induction (Figure 9). Induction of the bacteria at low initial OD values resulted in a low concentration of biomass. In contrast, induction of the bacterial culture containing a small number of cells using high IPTG concentration resulted in increased ectoine biosynthesis; thus, the amount of ectoine produced per cell dry weight (bacterial productivity) was higher.

Induction of the bacterial culture with a high concentration of inducer (>1.2 mM) and high initial OD values (>1.1) significantly decreased the productivity of the recombinant *E. coli* (Figure 8(b)) although the concentration of biomass was relatively low (Figure 9). These results indicate that the induction of bacterial culture at high initial OD values is less effective for gene expression and ectoine biosynthesis, resulting in low production of ectoine.

The response model predicted that the optimum concentration of extracellular ectoine (0.69 g/L) will be produced by the recombinant *E. coli* when the initial OD value is 0.74 and the final concentration of IPTG is 0.62 mM. Our experiments on those conditions enabled the recombinant cell to produce extracellular ectoine with an average concentration of 0.71 ± 0.03 g/L (data are shown in Supplementary Table 4). Additionally, the experimental data resulted in an average bacterial productivity of 375.9 ± 2.3 mg ectoine/g cdw, at optimum initial OD of 0.3 and IPTG concentration of 1.5 mM (data are shown in Supplementary Table 5). This result was consistent with the regression model, which predicted the maximum bacterial productivity of 374.8 mg ectoine/g cdw at the optimum initial OD and IPTG concentration. Compared to the first optimization which resulted in a maximum extracellular ectoine of around 0.37 g/L, this second optimization significantly increases the production of extracellular ectoine by the recombinant *E. coli*. This was due to the low initial OD value of the recombinant culture (around 0.5) and the low concentration of IPTG (0.5 mM) applied in the first optimization of ectoine production. As shown in Figure 8(a), induction of the recombinant cells at low initial OD value using a low concentration of IPTG decreased the yield of extracellular ectoine.

The optimization of ectoine production by the recombinant *E. coli*, using response surface methodology, successfully increased the concentration of extracellular ectoine from 0.23 to 0.71 g/L, an increment of nearly 3 times. Furthermore, the productivity of the recombinant bacteria was increased significantly from 69 to 376 mg ectoine/g cdw after optimization using RSM, an increment of more than 5 times. Other studies have reported the success of the response surface methodology in increasing ectoine production by halophilic bacteria [15, 18]. Thus, RSM is an effective method in the optimization of bioprocesses.

### 3.5. Production of Ectoine by the Recombinant *E. coli* against Incubation Time

**Figure 10:**
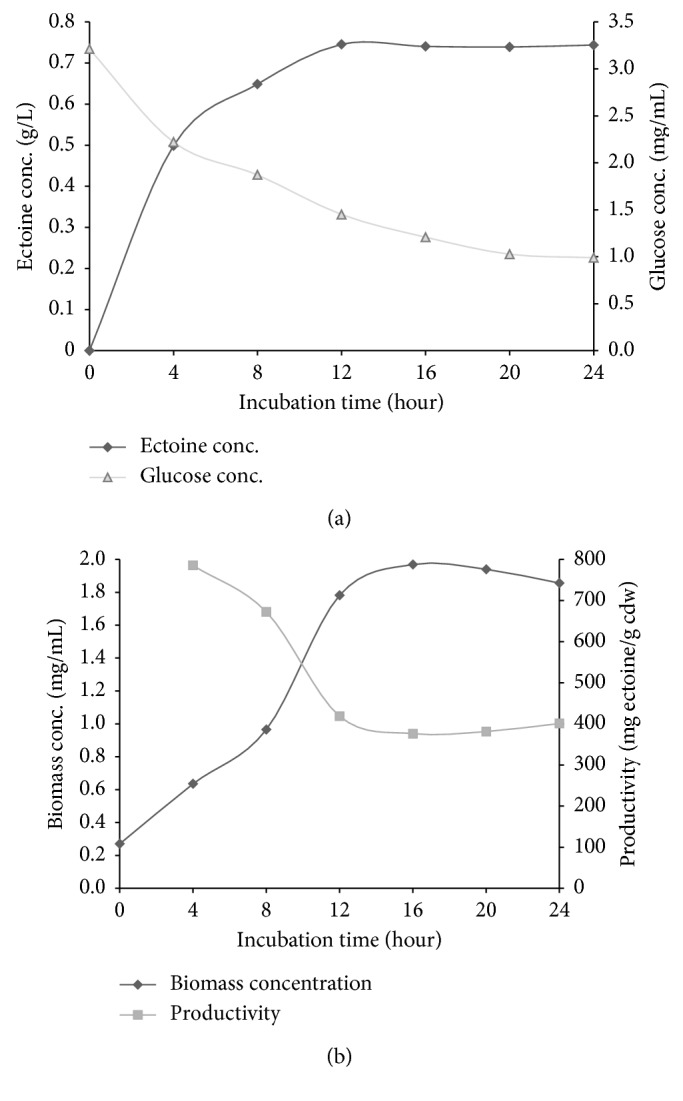
Production of ectoine by the recombinant *E. coli* against incubation time.

The production of ectoine by the recombinant *E. coli* was investigated at various incubation time periods. After reaching around 0.7 OD in the first cultivation, the subsequent incubation of the induced bacteria for 4 hours was able to produce about 0.5 g/L of extracellular ectoine (Figure 10(a)). Increasing the incubation time to 12 hours resulted in a significant increase in ectoine concentration. Herein, the recombinant cells produced the maximum concentration of ectoine of 0.75 g/L and remained constant during the longer incubation time. In contrast to the ectoine production, the level of glucose in the medium decreased steeply during the first 4 hours of cultivation and gradually slowed for the rest of the incubation time (Figure 10(a)). After 24 hours of incubation, the remaining glucose in the medium was about 1 mg/mL, while the glucose consumed by the bacteria was up by 90% relative to the initial concentration of 9.2 mg/mL.

The bacterial productivity was high during the first 4 hours of cultivation, at about 785 mg ectoine/g cdw, then decreased significantly until 12 hours of incubation, around 418 mg ectoine/g cdw, and finally stabilized for the rest of the incubation time (Figure 10(b)). In contrast, the biomass concentration was increased as the productivity of the bacteria was decreased (Figure 10(b)). This result indicates that the biosynthesis of ectoine was slowed down when the growth of the bacteria was accelerated. As a secondary metabolite, ectoine is generally produced optimally during the stationary phase, in which the growth of the bacteria is not dominant. Canovas [37] reported that the accumulation of ectoine in *Halomonas elongata* was optimum in the stationary phase. In addition, the transcription of ectoine biosynthesis genes was also optimum in the stationary phase [22]. However, the decrease in bacterial productivity at 12 hours and subsequent incubation did not influence the total concentration of extracellular ectoine produced, due to the increase in bacterial biomass concentration.

Ectoine produced by the recombinant *E. coli* BL21 in this study (0.75 g/L) was higher than what other researchers have reported. Expression of the ectoine gene cluster of *Bacillus halodurans* in *E. coli* strain M15, under the *T5* promoter, produced only about 4.6 mg/L of ectoine after 16 hours of incubation [10], lower than that produced by the recombinant *E. coli* in our study, which yielded around 0.75 g/L ectoine after 12 hours of incubation. Meanwhile, the productivity of *E. coli* DH5α harboring the ectoine gene cluster of *Marinococcus halophilus* was about 34 mg ectoine/g cdw [8], again lower than the productivity of the recombinant *E. coli* in our study (around 418 mg ectoine/g cdw). The productivity of our recombinant *E. coli* was also higher than the recombinant *E. coli* DH5*α* (around 5 mg/g cdw) used in [7], which expressed the ectoine gene cluster of *Chromohalobacter salexigens*. However, the concentration of ectoine after 160 hours of incubation was higher than our recombinant bacteria, i.e., about 6 g/L [7]. This was due to a higher cell density of the recombinant *E. coli*, about 22 g/L, compared to our recombinant bacteria of only around 1.8 mg/mL.

The yield of ectoine produced by the recombinant *E. coli* BK-AG25 in our study is lower than that reported in [9]. Using *E. coli* K-12 strain BW25113, expression of the ectoine gene cluster from *Halomonas elongata*, under arabinose-inducible promoter, was able to produce 2.67 mg/mL of extracellular ectoine after 24 hours of incubation. Bioconversion of ectoine by the recombinant *E. coli* K-12 was conducted using aspartate and glycerol as direct substrates [9]. Meanwhile, in our study, the production of ectoine was performed using indirect substrate, i.e., glucose as a carbon source for bacterial growth and ectoine biosynthesis. The low level of ectoine produced by the recombinant *E. coli* in our study was also due to the low expression of ectoine synthase (*ectC*), as revealed by SDS-PAGE. Thus, our future work is to elevate the expression of ectoine synthase, as it is a vital enzyme for the last step of ectoine biosynthetic pathway. This is the first report on the expression of an ectoine gene cluster of *Halomonas elongata* BK-AG25 in *E. coli* BL21 under the *T7* promoter. Overall, the productivity of the recombinant *E. Coli* in this study is the highest ever reported in terms of the use of indirect substrate for ectoine production. The optimization using RSM has significantly increased the production of ectoine by the recombinant cell.

## 4. Conclusion

Ectoine gene cluster of *Halomonas elongata* BK-AG25 was successfully expressed in *E. coli* under the *T7* promoter, in which most of the ectoines produced by the recombinant *E. coli* were excreted into the medium. Optimization of nutrients in the medium, as well as the bioprocess condition using response surface methodology, effectively increased the yield of ectoine and the productivity of the recombinant *E. coli*. After optimization, the recombinant cell was able to produce a high concentration of extracellular ectoine, about 0.75 g/L, after 12 hours of incubation, with bacterial productivity of around 418 mg ectoine/g cdw.

## Figures and Tables

**Table 1:** Experimental design for optimization of ectoine production by the recombinant *E. coli*.

Variable	Parameter	Level				
Equations (1) and (2)	Optimization I	−1.682	−1	0	1	1.682
*X*_1_	Glucose (% w/v)	0.3	0.6	1.0	1.4	1.7
*X*_2_	NaCl (% w/v)	0.1	0.6	1.4	2.2	2.7
*X*_3_	Temperature (°C)	15	21	30	39	45
Equations (3) and (4)	Optimization II	−1.414	−1	0	1	1.414
*X*_1_	OD before induction	0.3	0.4	0.8	1.1	1.2
*X*_2_	Final concentration of inducer (mM)	0.1	0.3	0.8	1.3	1.5

**Table 2:** Production of ectoine by the recombinant *E. coli*.

Experimental repetition	Biomass conc. (mg/mL)	Ectoine conc. (g/L)		Productivity (mg ectoine/g cdw)		Release of ectoine^*∗*^ (%)
		Intracellular	Extracellular	Intracellular	Extracellular	
1	3.5	0.074	0.22	21.4	63.6	75
2	3.3	0.070	0.23	21.0	70.3	77
3	3.4	0.070	0.25	20.7	72.9	78

Average standard deviation	3.4	0.071	0.23	21.0	68.9	77
	0.08	0.002	0.01	0.3	4.8	1.6

^*∗*^Release of ectoine is stated as the percentage of extracellular ectoine per total ectoine produced (extracellular + intracellular ectoines).

**Table 3:** Estimated coded coefficients and *p* values for ectoine production by recombinant *E. coli*, optimized using response surface methodology.

Factor	Coefficient	*p* value
Intracellular ectoine (g/L)		
Linear		
*X*_1_	0.00047	0.781
*X*_2_	0.00786	0.000^*∗*^
*X*_3_	−0.00015	0.921
Square		
*X*_1_^2^	−0.00140	0.402
*X*_2_^2^	−0.01084	0.000^*∗*^
*X*_3_^2^	−0.00978	0.000^*∗*^
Interaction		
*X*_2_ *X*_3_	0.01042	0.000^*∗*^

Extracellular ectoine (g/L)		
Linear		
*X*_1_	−0.0070	0.499
*X*_2_	−0.0717	0.000^*∗*^
*X*_3_	0.0356	0.004^*∗*^
Square		
*X*_1_^2^	−0.02001	0.061
*X*_2_^2^	−0.02468	0.025^*∗*^
*X*_3_^2^	−0.03795	0.002^*∗*^
Interaction		
*X*_2_ *X*_3_		

Productivity (mg ect./g cdw)		
Linear		
*X*_1_	2.81	0.121
*X*_2_	27.55	0.000^*∗*^
*X*_3_	−24.88	0.000^*∗*^
Square		
*X*_1_^2^	4.68	0.016^*∗*^
*X*_2_^2^	6.10	0.004^*∗*^
*X*_3_^2^	11.55	0.000^*∗*^
Interaction		
*X*_2_ *X*_3_	−12.10	0.000^*∗*^

^*∗*^Significant, *X*_1_ = glucose, *X*_2_ = NaCl, and *X*_3_ = temperature.

**Table 4:** Estimated coded coefficients and *p* values for ectoine concentration (g/L) and bacterial productivity (mg ectoine/g cdw), optimized using response surface methodology.

Factor	Coded coefficient	*p* value
Ectoine concentration (g/L)		
Linear		
*X*_1_	−0.0301	0.045^*∗*^
*X*_2_	−0.0313	0.039^*∗*^
Square		
*X*_1_^2^	−0.0556	0.004^*∗*^
*X*_2_^2^	−0.0398	0.020^*∗*^
Interaction		
*X*_1_ *X*_2_	−0.0736	0.004^*∗*^

Productivity (mg ectoine/g cdw)		
Linear		
*X*_1_	−37.88	0.000^*∗*^
*X*_2_	−7.64	0.182
Square		
*X*_1_^2^	−6.03	0.312
*X*_2_^2^	−12.80	0.054
Interaction		
*X*_1_ *X*_2_	−31.67	0.003^*∗*^

^*∗*^Significant, *X*_1_ = OD value before induction, *X*_2_ = the final concentration of inducer (IPTG).

## Data Availability

The data used to support the findings of this study are available from the corresponding author upon request.

## Supplementary Materials

Supplementary Materials Table 1: experimental and predicted values of intracellular ectoine concentration produced by the recombinant *E*. *coli*. Table 2: experimental and predicted values of extracellular ectoine concentration produced by the recombinant *E*. *coli*. Table 3: experimental and predicted values of the productivity of the recombinant *E*. *coli* producing intracellular ectoine. Table 4: experimental and predicted values of extracellular ectoine concentration produced by the recombinant *E*. *coli*. Table 5: experimental and predicted values of the productivity of the recombinant *E*. *coli* producing extracellular ectoine.
