# Supplementary material for: Heterologous Ectoine Production in Escherichia coli: Optimization Using Response Surface Methodology
Source: Int J Microbiol. 2019 Jul 1;2019:5475361. doi: 10.1155/2019/5475361 (PMC6636453; doi:10.1155/2019/5475361)
Supplement: Supplementary Materials — Table 1: experimental and predicted values of intracellular ectoine concentration produced by the recombinant E. coli. Table 2: experimental and predicted values of extracellular ectoine concentration produced by the recombinant E. coli. Table 3: experimental and predicted values of the productivity of the recombinant E. coli producing intracellular ectoine. Table 4: experimental and predicted values of extracellular ectoine concentration produced by the recombinant E. coli. Table 5: experimental and predicted values of the productivity of the recombinant E. coli producing extracellular ectoine. [file 5475361.f1.pdf]

Supplementary

Table 1. Experimental and predicted values of intracellular ectoine concentration produced by the recombinant *E. coli*

Run Order	Parameter			Ectoine concentration (g/L)		
	Glucose (% w/v)	NaCl (% w/v)	Temperature (°C)	Experiment	Prediction	Standard Error
1	0.3	1.4	30.0	0.045	0.053	0.0020
2	1.0	1.4	30.0	0.052	0.053	0.0020
3	1.0	1.4	30.0	0.053	0.053	0.0020
4	0.6	0.6	21.1	0.030	0.036	0.0033
5	0.6	2.2	38.9	0.053	0.051	0.0033
6	0.6	2.2	21.1	0.029	0.030	0.0033
7	1.0	1.4	15.0	0.031	0.026	0.0044
8	1.4	0.6	38.9	0.009	0.014	0.0033
9	1.4	0.6	21.1	0.037	0.036	0.0033
10	1.0	1.4	30.0	0.053	0.053	0.0020
11	0.6	0.6	38.9	0.025	0.014	0.0033
12	1.0	2.7	30.0	0.034	0.036	0.0044
13	1.0	1.4	30.0	0.056	0.053	0.0020
14	1.4	2.2	21.1	0.028	0.030	0.0033
15	1.4	2.2	38.9	0.056	0.051	0.0033
16	1.0	1.4	30.0	0.063	0.053	0.0020
17	1.0	1.4	30.0	0.050	0.053	0.0020
18	1.0	1.4	45.0	0.019	0.025	0.0044
19	1.0	0.1	30.0	0.009	0.009	0.0044
20	1.7	1.4	30.0	0.053	0.053	0.0020
1*	1.1	1.78	32	0.055	0.055	
2*	1.1	1.78	32	0.040	0.055	
3*	1.1	1.78	32	0.055	0.055	
			Average :	0.050		
			Standard error :	0.005		

*Expression and production of intracellular ectoine by the recombinant *E. coli* at the optimum value of glucose and NaCl levels and incubation temperature.

Table 2. Experimental and predicted values of extracellular ectoine concentration produced by the recombinant *E. coli*

Run Order	Parameter			Ectoine concentration (g/L)		
	Glucose (% w/v)	NaCl (% w/v)	Temperature (°C)	Experiment	Prediction	Standard Error
1	0.3	1.4	30.0	0.264	0.230	0.029
2	1.0	1.4	30.0	0.312	0.274	0.015
3	1.0	1.4	30.0	0.328	0.274	0.015
4	0.6	0.6	21.1	0.229	0.235	0.020
5	0.6	2.2	38.9	0.117	0.163	0.020
6	0.6	2.2	21.1	0.114	0.091	0.020
7	1.0	1.4	15.0	0.086	0.107	0.029
8	1.4	0.6	38.9	0.341	0.292	0.020
9	1.4	0.6	21.1	0.211	0.221	0.020
10	1.0	1.4	30.0	0.243	0.274	0.015
11	0.6	0.6	38.9	0.270	0.306	0.020
12	1.0	2.7	30.0	0.097	0.084	0.029
13	1.0	1.4	30.0	0.244	0.274	0.015
14	1.4	2.2	21.1	0.100	0.078	0.020
15	1.4	2.2	38.9	0.121	0.149	0.020
16	1.0	1.4	30.0	0.252	0.274	0.015
17	1.0	1.4	30.0	0.265	0.274	0.015
18	1.0	1.4	45.0	0.259	0.227	0.029
19	1.0	0.1	30.0	0.323	0.325	0.029
20	1.7	1.4	30.0	0.182	0.206	0.029
1*	0.92	0.28	34	0.31	0.34	
2*	0.92	0.28	34	0.34	0.34	
3*	0.92	0.28	34	0.40	0.34	
4*	0.92	0.28	34	0.44	0.34	
			Average :	0.37		
			Standard error :	0.027		

*Expression and production of extracellular ectoine by the recombinant *E. coli* at the optimum value of glucose and NaCl levels and incubation temperature.

Table 3. Experimental and predicted values of the productivity of the recombinant *E. coli* producing intracellular ectoine

Run Order	Parameter			Productivity (mg ectoine/g cdw)		
	Glucose (% w/v)	NaCl (% w/v)	Temperature (°C)	Experiment	Prediction	Standard Error
1	0.3	1.4	30.0	43.3	40.2	4.8
2	1.0	1.4	30.0	29.3	31.7	2.5
3	1.0	1.4	30.0	31.7	31.7	2.5
4	0.6	0.6	21.1	34.2	34.9	5.0
5	0.6	2.2	38.9	42.2	43.3	5.0
6	0.6	2.2	21.1	109.9	105.8	5.0
7	1.0	1.4	15.0	98.4	106.2	4.8
8	1.4	0.6	38.9	5.2	6.5	5.0
9	1.4	0.6	21.1	47.5	43.6	5.0
10	1.0	1.4	30.0	32.6	31.7	2.5
11	0.6	0.6	38.9	15.6	20.8	5.0
12	1.0	2.7	30.0	88.0	95.3	4.8
13	1.0	1.4	30.0	33.2	31.7	2.5
14	1.4	2.2	21.1	139.4	131.3	5.0
15	1.4	2.2	38.9	49.4	45.8	5.0
16	1.0	1.4	30.0	35.7	31.7	2.5
17	1.0	1.4	30.0	28.3	31.7	2.5
18	1.0	1.4	45.0	26.3	22.5	4.8
19	1.0	0.1	30.0	6.0	2.6	4.8
20	1.7	1.4	30.0	42.6	49.6	4.8

Table 4. Experimental and predicted values of extracellular ectoine concentration produced by the recombinant *E. coli*

Run Order	Parameter		Ectoine Concentration (g/L)		
	OD before induction	IPTG (mM)	Experiment	Prediction	Standard Error
1	0.4	1.3	0.68	0.66	0.028
2	0.8	0.1	0.68	0.65	0.028
3	1.1	0.3	0.64	0.66	0.028
4	0.4	0.3	0.55	0.58	0.028
5	0.8	0.8	0.68	0.68	0.016
6	0.8	0.8	0.66	0.68	0.016
7	0.8	0.8	0.73	0.68	0.016
8	0.8	0.8	0.66	0.68	0.016
9	1.1	1.3	0.48	0.45	0.028
10	0.8	0.8	0.69	0.68	0.016
11	1.2	0.8	0.53	0.53	0.028
12	0.3	0.8	0.62	0.62	0.028
13	0.8	1.5	0.53	0.56	0.028
1*	0.74	0.62	0.71	0.69	
2*	0.74	0.62	0.77	0.69	
3*	0.74	0.62	0.64	0.69	
4*	0.74	0.62	0.73	0.69	
		Average :	0.71		
		Standard error :	0.03		

*Expression and production of extracellular ectoine by the recombinant *E. coli* at the optimum value of OD before induction and the final concentration of IPTG.

Table 5. Experimental and predicted values of the productivity of the recombinant *E. coli* producing extracellular ectoine

Run Order	Parameter			Productivity (mg ectoine/g cdw)		
	OD before induction	IPTG (mM)		Experiment	Prediction	Standard Error
1	0.4	1.3		363.1	349.4	11.5
2	0.8	0.1		291.8	291.6	11.5
3	1.1	0.3		279.3	289.0	11.5
4	0.4	0.3		312.0	301.4	11.5
5	0.8	0.8		318.3	306.4	6.5
6	0.8	0.8		301.5	306.4	6.5
7	0.8	0.8		320.8	306.4	6.5
8	0.8	0.8		293.9	306.4	6.5
9	1.1	1.3		203.7	210.3	11.5
10	0.8	0.8		297.3	306.4	6.5
11	1.2	0.8		253.1	240.7	11.5
12	0.3	0.8		331.5	347.9	11.5
13	0.8	1.5		265.8	270.0	11.5
1*	0.3	1.5		372.7	374.8	
2*	0.3	1.5		379.3	374.8	
3*	0.3	1.5		380.5	374.8	
4*	0.3	1.5		371.1	374.8	
		Average	:	375.9		
		Standard error	:	2.3		

*Expression and production of extracellular ectoine by the recombinant *E. coli* at the optimum value of glucose and NaCl levels and incubation temperature.
